# Comprehensive analysis of angiogenesis subtype of squamous cell carcinoma

**DOI:** 10.1186/s12957-021-02367-3

**Published:** 2021-09-14

**Authors:** Fanglu Qin, Shenghua Lin, Kun Deng, Junqi Qin, Zhanyu Xu, Liqiang Yuan, Jiangbo Wei, Yu Sun, Tiaozhan Zheng, Shikang Li

**Affiliations:** 1 Department of Thoracic and Cardiovascular Surgery, The First Affiliated Hospital of Guangxi Medical University, Nanning, 530021 Guangxi Zhuang Autonomous Region People’s Republic of China; 2 Guangxi Medical University, Nanning, 530021 Guangxi Zhuang Autonomous Region People’s Republic of China; 3 Department of Thoracic and Cardiovascular Surgery, People’s Hospital of Guangxi Zhuang Autonomous Region, Nanning, 530021 Guangxi Zhuang Autonomous Region People’s Republic of China

**Keywords:** Squamous cell carcinoma, Angiogenesis, TCGA, Comprehensive analysis

## Abstract

**Background:**

Squamous cell carcinoma (SCC) is a disease with distinct management complexities as it displays a remarkably heterogeneous molecular subtype. However, the landscape of angiogenesis for SCC is not fully investigated.

**Method and materials:**

The angiogenesis-related subtypes of SCC were established by using the ConsensusClusterPlus package based on angiogenesis-related genes and TCGA data. We analyzed the alteration of genes and miRNAs as well as pathways associated with angiogenesis subtypes. Next, the regulation network, the correlation with genomic characteristics, immune microenvironment, and clinical features of the angiogenesis subtypes were further investigated. Finally, the prognostic impact of the angiogenesis-related subtypes for SCC was also analyzed.

**Results:**

A total of 1368 SCC samples were included in this study. Two angiogenesis subtypes were then identified based on the one hundred and sixty-three angiogenesis-related genes with subtype1 (angiogenesis subtype) of 951 SCC patients and subtype2 (non-angiogenesis subtype) of 417 SCC. GSEA revealed that angiogenesis and epithelial-mesenchymal transition, inflammatory response, and hypoxia were enriched in the angiogenesis subtype. Eight of the 15 immune checkpoints (ADORA2A, BTLA, CD276, CYBB, HAVCR2, SIGLEC7, SIGLEC9, and VTCN1) were significantly upregulated while C10orf54 were significantly downregulated in the angiogenesis subtype. The survival analysis revealed that the patients in the angiogenesis subtype have poorer survival outcomes than those in the non-angiogenesis subtype (*P* = 0.017 for disease-free interval and *P* = 0.00013 for overall survival).

**Conclusion:**

Our analysis revealed a novel angiogenesis subtype classification in SCC and provides new insights into a hallmark of SCC progression.

## Introduction

Squamous cell carcinoma (SCC) represents the most common human solid tumor and is a major cause of cancer mortality [1]. The occurrence of SCC is closely related to the key changes in genome disturbance, gene mutation, and molecular expression at different stages. Fundamental changes in mesenchymal cells also play an important role in the development of these tumors, which might even be a major determinant of promoting immune escape and chemotherapy drug resistance [2]. PAM50, a classification of breast cancer widely used in gene expression profiling, can divide different clinical outcomes into five subtypes [3]. On the other hand, changes in certain cancer-related genes epidermal growth factor receptor (EGFR) and TP53 pathway present in different SCC types [4, 5]. Also, it is demonstrated that the smoking status is an important factor for the response of immune therapy for SCC like non-small cell lung cancer (NSCLC) [6]. The novel roles of the protein-coding genes were found in SCC regarding angiogenesis and aerobic glycolysis pathway [7–10]. The recent studies also focus on miRNA [11] and lncRNAs [12] involved in the tumorigenesis and progression of SCC. SCCs share common histologic features and have similar molecular patterns, which are different from other cancer types [13].

Angiogenesis is a change in the balance between pro-angiogenesis and anti-angiogenesis factors [14]. Increased angiogenesis is associated with tumor progression, metastasis, and unfavorable outcome [15]. Numerous studies have shown that solid tumors are “angiogenesis-dependent” [16]. For example, in oral squamous cell carcinoma (OSCC), keratinocytes, and inflammatory cells directly produce a variety of molecules that can induce angiogenesis. Besides, in many different tumors including head and neck squamous cell carcinoma (HNSCC), the increased expression of vascular endothelial growth factor (VEGF) protein contributes to the induction of angiogenesis in tumors [17, 18]. The relationship between angiogenesis and tumor is a recognized factor, while the full biological mechanisms on angiogenesis and SCC are not fully investigated. Although in recent years it has made many pan-cancer classifications based on gene expression, a consensus on angiogenesis molecular for SCC was not yet established. Therefore, it is essential to discover the new molecular subtypes of SCC research.

To determine the association between SCC subtypes and the angiogenesis process, this study established a novel SCC classification based on 474 angiogenesis genes and The Cancer Genome Atlas (TCGA) database. The regulation network, genome mutations, immune characteristics, and prognostic value of subtypes were explored to unveil the potential associations between angiogenesis and SCC.

## Materials and methods

### Data download and preprocessing

TCGA pan-cancer data were downloaded from the UCSC Genome Browser (https://genome.ucsc.edu), including batch-effect normalized transcription data, clinical data, single-sample gene set enrichment analysis (ssGSEA) score data, drug target data, homologous recombination deficiency (HRD) score and genome-wide DNA damage data, immune signature scores data, and RNA-based stemness scores data. All data processing is described on the official website. The pan-cancer study combined with clinical data included a total of 1368 SCC samples, including 252 cervical squamous cell carcinomas (CESCs), 95 esophageal squamous cell carcinomas (ESCAs), 520 HNSCs, and 501 lung squamous cell carcinomas (LUSCs).

### Angiogenesis subtypes

First of all, we obtained 507 angiogenesis-related genes from the AmiGO2 website (http://amigo.geneontology.org/amigo). Combined with gene expression data, 474 angiogenesis genes were finally obtained for analysis. Univariable Cox analysis was used to filter the angiogenesis genes which had the prognostic value for SCC patients (*P* < 0.05). Based on the prognostic angiogenesis-related genes, ConsensusClusterPlus R-package was used to identify subtypes in SCC tumor samples using 1000 iterations, 80% sample resampling from 2 to 7 clusters (k2 to k7) using kmdist with average linkage algorithm and correlation as the similarity metric.

### Gene set enrichment analysis and pathway

To study the changes in gene sets, Gene Set Enrichment Analysis (GSEA) was performed on all genes [19]. We analyzed the correlations between angiogenesis subtypes with cancer hallmark pathways and the Kyoto Encyclopedia of Genes and Genomes (KEGG) pathway in each tumor sample. GSEA can highlight genes associated with the subtypes through pathway analysis. Gene Ontology (GO) analysis and KEGG pathway enrichment analysis were also performed based on differentially expressed genes (DEGs).

### Genomic correlations with angiogenesis subtypes

Aneuploidy and LOH scores and ABSOLUTE purity/ploidy files were obtained from the research by Thorsson et al. [20]. All purity, ploidy, LOH, and copy number variant (CNV) invocation used to create the DNA damage scores were derived by the TCGA Aneuploidy AWG using ABSOLUTE [21]. Moreover, HRD and HRD-loss of heterozygosity (HRD-LOH) scores were obtained from the UCSC genome browser. The copy number burden fraction change and the number of segments represent the base fraction deviating from the baseline multiplicity and the total number of segments in each sample’s copy number profile, respectively. Each fragment was designated as amplification, deletion, or neutral based on its number of copies relative to the circular ploidy of the sample. In addition, we calculated Oncoplot, mutation landscape, and OncogenicPathways based on TCGAmutation and maftools R packages.

### Differentially expressed genes and regulations associated with angiogenesis

Mann-Whitney *U* test was performed to derive the DEGs and miRNAs between subtype1 and subtype2 (FDR < 0.05, absolute logFC > 1). Abnormal vascular networks due to tumor cells can secrete a large number of pro-angiogenesis factors, which are characterized by vascular disease, immaturity, and permeability [22]. To clarify the regulation of angiogenesis subtypes, we performed a computational analysis to identify two “master regulators”: transcription factors (TF) and miRNA. We first downloaded 318 TFs from the Cistrome Bowser (http://cistrome.org/). The correlation was analyzed between 163 angiogenesis genes obtained from survival analysis with TF (person correlation: *R*^2^ = 0.4, *P* < 0.05). The Mann-Whitney *U* test was utilized to calculate the differential miRNA (FDR = 0.05, log FC > 1). And the target gene of five miRNAs was predicted by TargetScan (http://www.targetscan.org/vert_72/). Each of the predicted miRNA target genes is the result of the correlation test which intersected angiogenesis genes. Based on TransmiR v2.0 database (http://www.cuilab.cn/transmir), the predicted miRNA was then intersected with the TF. Finally, the TF-miRNA-target gene regulatory network was constructed using Cytoscape software (https://cytoscape.org/).

### The microenvironment in the angiogenesis subtypes

Tumor-infiltrating leukocytes (TILs) have been shown to be associated with tumor prognosis and treatment response and are a crucial component of the tumor microenvironment [23]. CIBERSORT is a common computational method for the quantification of cellular components by gene expression profiling (GEP) from bulk tissues [24]. Therefore, we upload the gene expression data to the CIBERSORT website, which can be obtained free of charge (https://cibersort.stanford.edu/). We used a leukocyte gene signature matrix, termed LM22 and 1000 permutations. LM22 contains 547 genes and distinguishes 22 kinds of human hematopoietic cell subtypes, including 7 types of T cells, naive and memory B cells, plasma cells, NK cells, and myeloid subpopulations [23]. We rank the gene expression values of a given sample by ssGSEA and then calculated the enrichment score of angiogenesis genes in each sample. In the ssGSEA analysis, we used only genes associated with angiogenesis for our calculations by GSVA, limma, and GSEABase R packages. The Estimating Stromal Cells and Immune Cells in Malignant Tumor Tissues Using Expression Data (ESTIMATE) algorithm was used to calculate stromal cells, immune cells, and estimated scores [25].

### Statistical analysis

The survival outcome of these patients with different subtypes was calculated by Log-rank test. To investigate the correlation between clinicopathological characteristics and SCC, we further studied the relationship between angiogenesis subtypes and gender, clinical stage (I~IV), tumor status, and histological grade by Chi-square test. Pearson correlation test was performed to evaluate the immune signatures level for each sample. The enrichment levels of 68 immune signatures and angiogenesis were quantified by the heatmap R package. We compared the differences in drug target types between subtype1 and subtype2 and visualized the differences using the “pheatmap” R software package. A two-sided *P* value less than 0.05 was set as a statistical significance threshold. All the analysis were performed based on R version 3.4.2 (https://www.r-project.org/).

## Results

### Angiogenesis subtypes

**Fig. 1:**
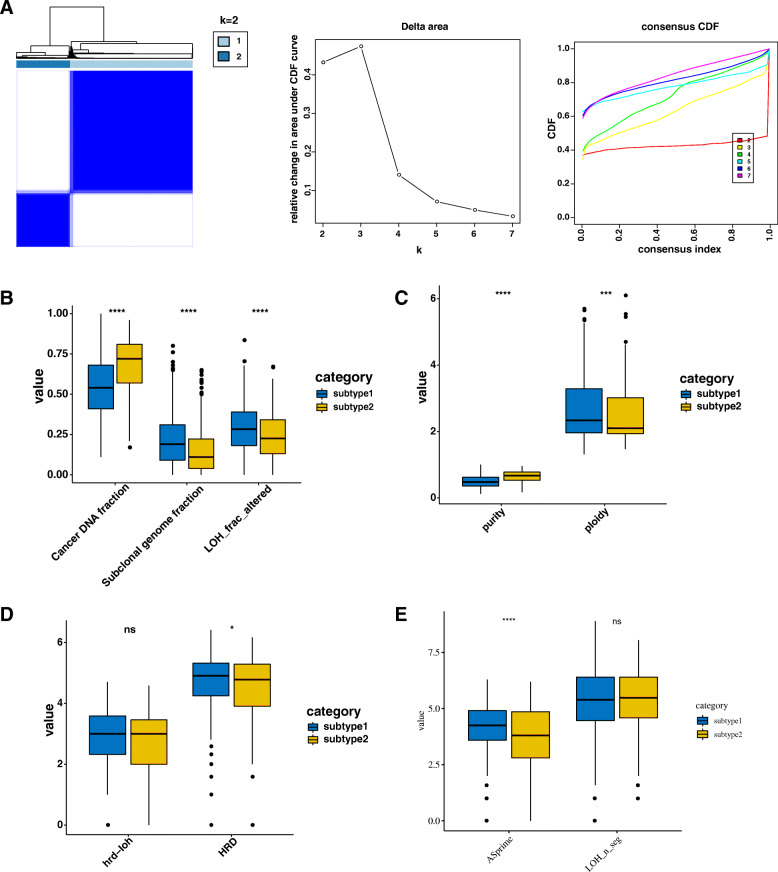
Clusters and genomic alteration of different subtypes in SCC. **A** Consensus cluster for SCC patients based on prognostic angiogenesis genes. **B** DNA damage scores of different subtypes of SCC. **C** Tumor purity and ploidy of different subtypes of SCC. **D** Homologous recombination deficiency (HRD) and HRD-loss of heterozygosity (HRD-LOH) scores. **E** Prime loss of heterozygosity and fractions in different subtypes of SCC

A total of 252 CESCs, 95 ESCAs 520 HNSCs, and 501 LUSCs were included in this study. A total of 90 grade I, 461 grade II, 248 grade III, and 8 grade IV SCC were analyzed (Table 1). Based on univariate Cox analysis for survival analysis, we got one hundred and sixty-three genes with the prognostic impact on SCC (*P* < 0.05). When *K* = 2, the module boundaries are clearest and free of crossings. We divided the patients into subtype1 (951 SCC patients) and subtype2 (417 SCC patients) based on the one hundred and sixty-three genes of survival analysis (Fig. 1A).

**Table 1:** Characteristic of SCC patients

Characteristic	TCGA cohort (***n*** = 1368)	Subtype1 cohort (***n*** = 951)	Subtype2 cohort (***n*** = 417)
**Age**			
≤ 41	108	41	67
> 41	1250	900	350
NA	10	10	0
**Gender**			
Male	836	656	180
Female	532	295	237
**Cancer type**			
CESC	252	60	192
ESCA	95	74	21
HNSC	520	420	100
LUSC	501	397	104
**Grade**			
Grade I	90	66	24
Grade II	461	313	148
Grade III	248	135	113
Grade IV	8	6	2
NA	561	431	130
**Clinical stage**			
Stage I	146	44	102
Stage II	186	116	70
Stage III	160	104	56
Stage IV	303	235	68
NA	573	452	121
**Tumor status**			
Tumor free	840	597	276
With tumor	528	258	114
NA	123	96	27

### Genomic correlations with angiogenesis subtypes

**Fig. 2:**
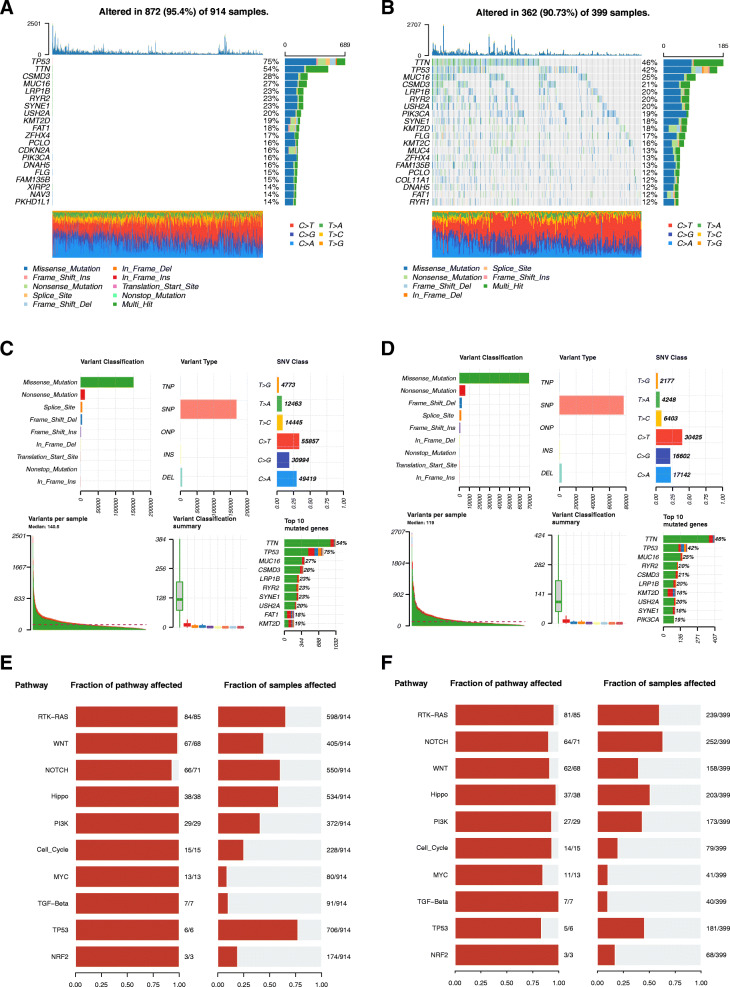
Mutations between two subtypes. **A** The ratio of the top 20 genes in the number of mutations in subtype1. **B** The ratio of the top 20 genes in the number of mutations in subtype2. **C** The landscape of mutation panorama for the number of mutations and mutation types in subtype1. **D** The landscape of mutation panorama for the number of mutations and mutation types in subtype2. **E** The number of mutated genes contained in the number of mutated samples per pathway in subtype1. **F** The number of mutated genes contained in the number of mutated samples per pathways in subtype2

As result, the cancer DNA fraction, subclonal genome fraction, and LOH fraction altered were significantly higher in subtype1 (Fig. 1B). Additionally, the tumor purity was significantly lower and the ploidy was significantly higher in subtype1 (Fig. 1C). However, the HRD-LOH presented no significant difference between the two subtypes. HRD in subtype1 was significantly higher than that in subtype2 (Fig. 1D). The more prime LOH was found in subtype1 (Fig. 1E). Moreover, the first 20 highly mutated genes were shown in Oncoplot (Fig. 2A, B). A landscape of numbers of mutations and mutation types was showed in Fig. 2C and D. Ten significantly enriched oncogenic pathways had the greatest impact in subtype1 including RTK-RAS, WNT, Hippo, PI3K, Cell Cycle, MYC, TGF-Beta, TP53, and NRF2 (Fig. 2E). The TGF-Beta and NRF2 pathways had the greatest effect in subtype2 (Fig. 2F).

### Differentially expressed genes and network regulation of angiogenesis subtypes

**Fig. 3:**
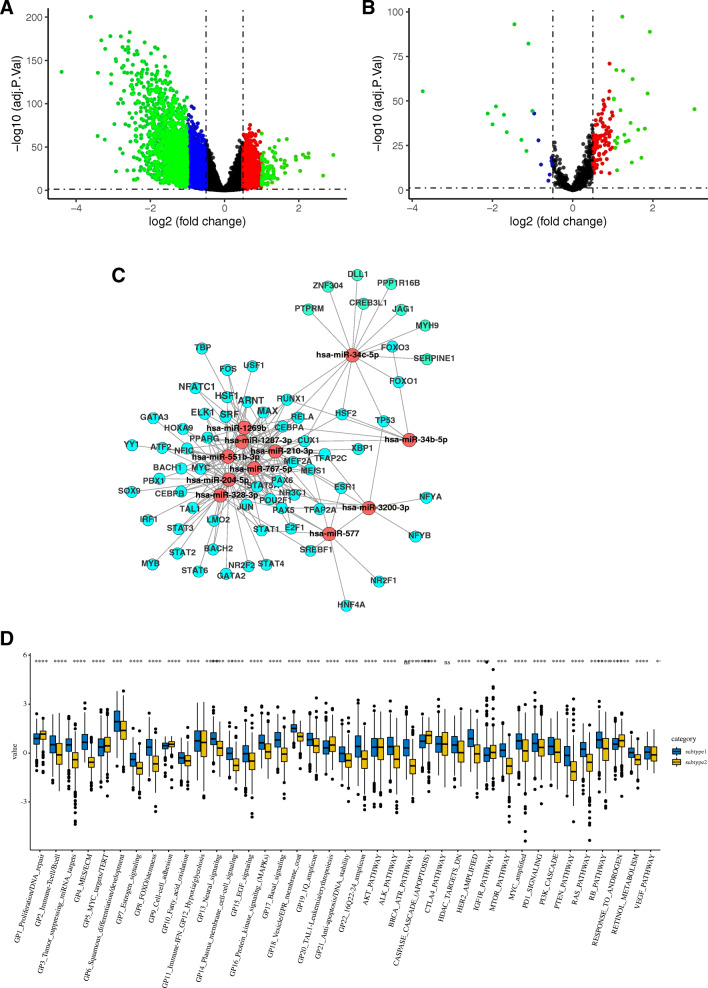
The regulation of genes and networks of angiogenesis subtype. **A** Differently expressed genes between angiogenesis subtype and non-angiogenesis subtype. **B** Differently expressed miRNAs between angiogenesis subtype and non-angiogenesis subtype. **C** The regulation network of genes-miRNAs-TFs that were altered by angiogenesis

We identified 163 DEGs including 124 upregulated genes and 18 with downregulated genes in the subtype1 (*P* < 0.05, Fig. 3A). Next, we analyzed the differently expressed miRNAs between the two subtypes. As result, a total of 34 differently expressed miRNAs were found between two subtypes, including 11 downregulated miRNAs and 23 upregulated miRNAs (Fig. 3B). To reveal whether the regulatory relationship between TF and miRNAs has an impact on SCC, we established the regulation network for TF-miRNA-target (Fig. 3C). Finally, most of the drug targets were shown to be also altered by angiogenesis (Fig. 3D).

### Enrichment pathways for angiogenesis subtype

**Fig. 4:**
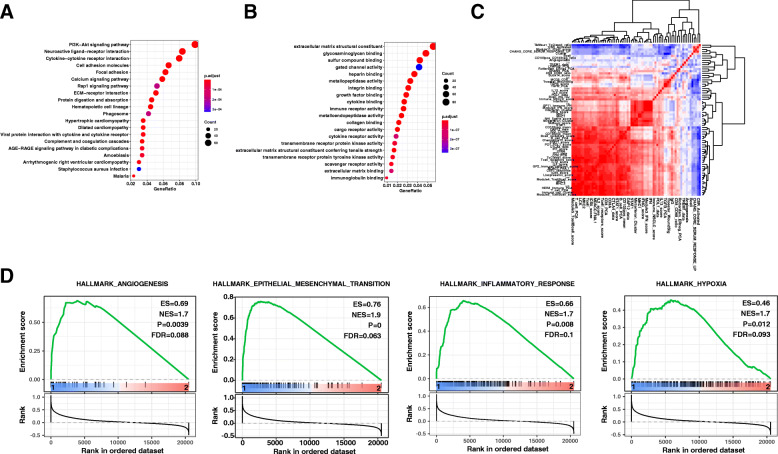
Alteration of pathways for the different subtypes of SCC. **A** KEGG pathway analysis associated with DEGs. **B** GO pathway analysis associated with DEGs. **C** The correlation of different pathways and angiogenesis subtype. **D** Four hallmark pathways of cancer enriched in angiogenesis subtype

According to DEGs, twenty pathways were significantly enriched (*P* < 0.05) by performing KEGG pathway analysis, which included the PI3K/Akt signaling pathway and ECM receptor interactions (Fig. 4A). Notably, twenty GO terms with DEGs were also found to be enriched, extracellular matrix structural constituent with the most significant P-value, with the maximum difference (Fig. 4B). Figure 4C shows the correlation between different pathways and the angiogenesis subtype. GSEA analysis shows these pathways were strongly associated with angiogenesis in tumor progression (Fig. 4D). Thus, we defined subtype1 as the angiogenesis subtype and subtype2 as the non-angiogenesis subtype. As for the angiogenesis subtype, ten pathways were found to be upregulated including focal adhesion, pathways in cancer, adherens junction, renal cell carcinoma, regulation of actin cytoskeleton, neurotrophin signaling pathway, ECM receptor interaction, TGF-beta signaling pathway, ERBB signaling pathway, and GAP junction. On the other hand, another ten KEGG pathways were upregulated in the non-angiogenesis subtype such as spliceosome, base excision repair DNA replication. Notably, ten hallmarks’ pathways were also found to be enriched in the angiogenesis subtype, including epithelial-mesenchymal transition, inflammatory response, hypoxia, and angiogenesis. As for subtype2, oxidative phosphorylation DNA repair, E2F targets, MCY targets v1, G2M checkpoint, and MYC targets v2 were found to be the enriched hallmarks pathways.

### The immune microenvironment in the angiogenesis subtypes

**Fig. 5:**
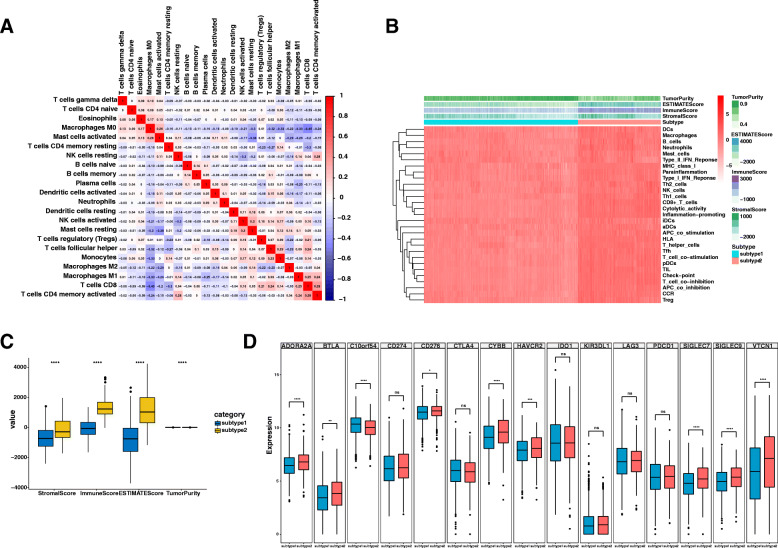
The immune microenvironment of angiogenesis subtype. **A** Associations between 22 different leukocyte subsets and angiogenesis subtype. **B** Expression of immune cells between angiogenesis subtype by ssGSEA. **C** The ESTIMATE score immune score, stromal score, and tumor purity between the angiogenesis subtype and the non-angiogenesis subtype. **D** Expression of immune checkpoints for the angiogenesis subtype

Next, we aimed to explore the microenvironment in these two angiogenesis subtypes. By applying CIBERSORT, we discovered complex associations between 22 different leukocyte subsets and angiogenesis subtypes (Fig. 5A). We also explored the distribution of the immune cells between the angiogenesis subtype and non-angiogenesis subtype (Fig. 5B). Additionally, we also evaluated the immune score by using the ESTIMATE algorithm. As result, the ESTIMATE score immune score and stromal score were significantly higher in the angiogenesis subtype and the tumor purity was significantly lower in the non-angiogenesis subtype (Fig. 5C). Further, we analyzed the expression of 15 immune checkpoints between two angiogenesis subtypes. The results showed that 8 of the 15 immune checkpoints (ADORA2A, BTLA, 276, CYBB, HAVCR2, SIGLEC7, SIGLEC9, and VTCN1) were significantly upregulated while C10orf54 were significantly downregulated in the angiogenesis subtype (Fig. 5D).

### The clinical implication of angiogenesis subtypes

**Fig. 6:**
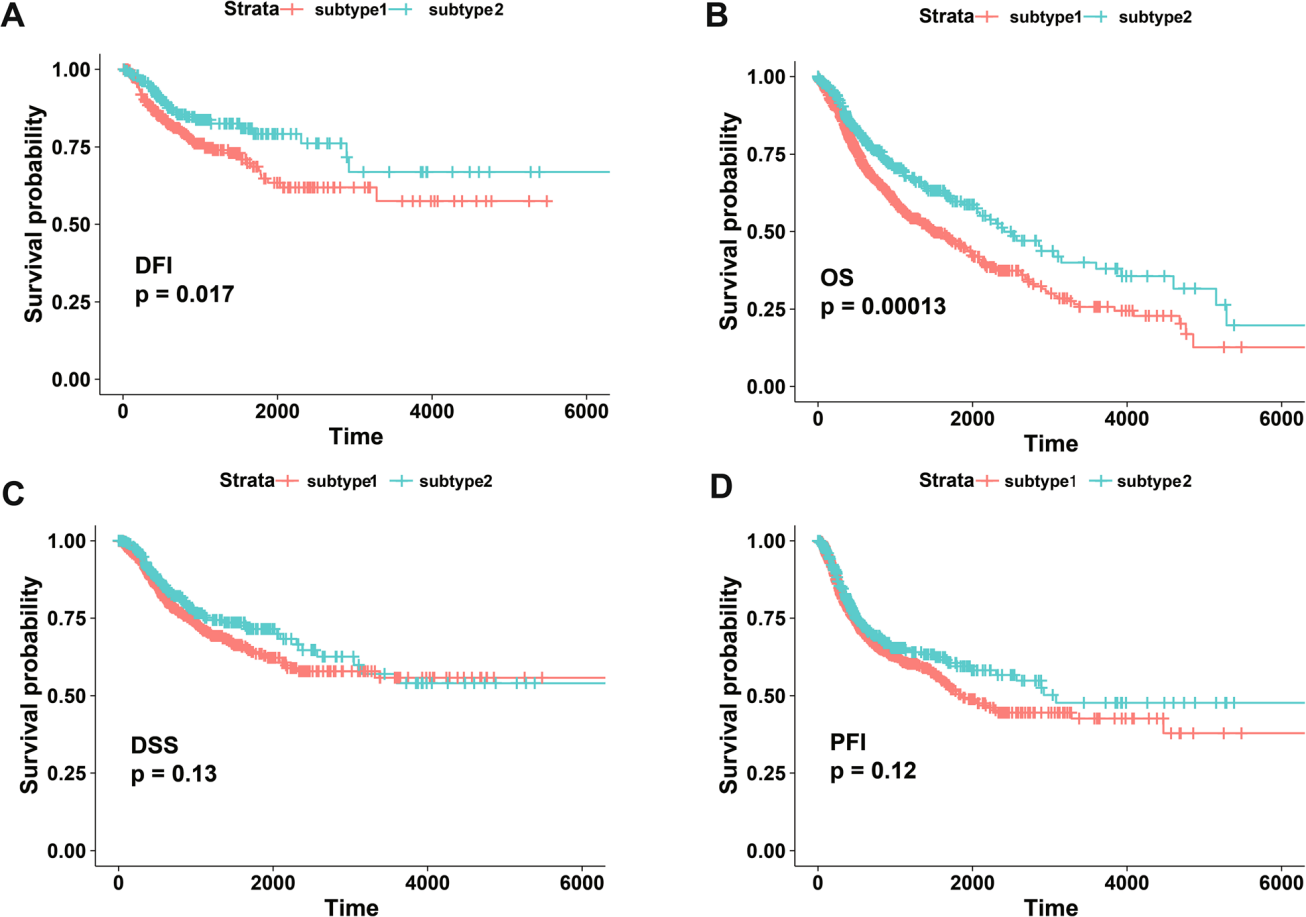
Prognostic of angiogenesis subtype for SCC patients. **A** Disease-free interval (DFI). **B** Overall survival (OS). **C** Disease-specific survival (DSS). **D** Progression-free interval (PFS)

To evaluate the prognostic significance of angiogenesis subtype for SCC patients. The KM curves revealed that the patients in the angiogenesis subtype have a lower DFI than that in the non-angiogenesis subtype (*P* = 0.017, Fig. 6A). Additionally, the patients of the angiogenesis subtype were revealed to have a poor OS outcome than that of the non-angiogenesis subtype (*P* = 0.00013, Fig. 6B). However, there was no statistical significance was found for the DSS and PFI (Fig. 6C, D).

### Discussion

In this study, we represent the novel insights into the angiogenic features in 1368 samples from 4 SCC types, in which two novel angiogenesis-related subtypes of SCC were established based on TCGA database. The angiogenesis subtype displayed distinct angiogenesis characteristics and had a poorer prognosis, providing new evidence for the progression of SCC.

So far, most studies investigating the subtypes and SCC were mainly focused on the immune microenvironment. A previous pan-cancer study classified the SCCs fell into wound healing and IFN g dominant subtypes based on the immune signature [20]. Another TCGA study has identified six immune subtypes of SCCs including angiogenesis, while the molecular characteristics of angiogenesis were not fully uncovered [26]. Recently, Benjamin et al. developed an endothelial index to estimate the vascular density of 31 solid tumor types and classified the human tumors into 6 vascular microenvironment signatures based on the 24 vascular “hub” genes [27]. While the different histology types of tumors present widely different patterns, our study only performed the analysis for SCCs and aimed to find out similar features for these types of tumors, providing the evidence for the personalized therapy.

Angiogenesis is a complex process that plays a vital role in organ and tissue regeneration, growth and development, and many pathological conditions [28]. Angiogenesis contributes to the development of malignant subtype traits. It is believed that the transition to the angiogenesis subtype is caused by the change in the balance of positive and negative regulators of angiogenesis [29]. Tumors require a blood supply to grow and may be generated by the expression of pro-angiogenesis growth factors. Our study revealed that the angiogenesis subtype was enriched in the epithelial-mesenchymal transition (EMT) pathway. EMT has been closely linked to “stemness” in the development of cancer [30]. The study by Zhang et al. showed that endothelial cells secrete factors which may be attracted to the epithelial tumor cells by blood vessels, allowing it to pass through the EMT connective tissue transfer, and to enhance its potential by imparting tumorigenic tumor cells with a stem cell-like phenotype [31]. Ke et al. found that VEGF-A and Notch signaling pathways were activated by LncRNA NEAT1, leading to promote EMT and repress apoptosis in oral squamous cell carcinoma [12]. Moreover, the results and also the gene set in inflammatory were enriched in the angiogenesis subtype. Inflammatory responses increase the risk of developing many types of cancer. The hallmark of cancer-related inflammation includes inflammatory cells and tissue expression of inflammatory mediators (e.g., chemokines, cytokines, and prostaglandins), and tissue remodeling, and angiogenesis, chronic inflammatory diseases, and similar reactions and tissue repair [32]. Furthermore, hypoxia is a key factor in the tumor angiogenesis process. Studies have shown that tissue is upregulated by HIF-1a promoter steady-state reaction, to adapt to hypoxia, which may further facilitate tumor growth and tumor angiogenesis [33]. In summary, the angiogenesis subtype is linked to the characteristics of angiogenesis closely and promotes the development of cancer.

Also, eight of the 15 immune checkpoint genes were upregulated, whereas C10orf54 was significantly downregulated in the angiogenesis subtype. Cancer cells have various mechanisms to evade the local immune attack, including upregulation of immune checkpoint proteins [34]. Immune checkpoint proteins are activated by ligand-receptor, resulting in a dynamic balance between stimulatory and non-stimulatory, and inhibitory signals that regulate the immune response [35]. This phase of equilibrium is ensured primarily by the PD-1/PD-L pathway, which inhibits the activation and proliferation of T-cell as well as cytokine production. And the cytotoxic T-lymphocyte-associated antigen 4 (CTLA-4) pathway, which induces cell cycle arrest and apoptosis in Tregs and T cells activation. However, once tumor antigens expressed by tumor cells can bypass the immune system, the balance is altered [34, 36] and the final result showed the progression of cancerous tumors in clinical practice [37]. The appropriate subtype classification may provide evidence for the selection of appropriate therapy.

## Conclusions

In summary, we established a novel subtype classification related to angiogenesis for SCC with distinct molecular characteristics and clinical implications. This work provides new sights into the progression of SCC, contributing to the future design of personalized therapy.

## Data Availability

Data and materials of this work are available from the corresponding author on reasonable request.

## Abbreviations

SCC: Squamous cell carcinoma
OSCC: Oral squamous cell carcinoma
HNSC: Head and neck squamous cell carcinoma
ssGSEA: Single-sample gene set enrichment analysis
CESC: Cervical squamous cell carcinoma
ESCA: Esophageal squamous cell carcinomas
LUSC: Lung squamous cell carcinoma
HRD: Homologous recombination deficiency
GSEA: Gene set enrichment analysis
KEGG: Kyoto encyclopedia of genes and genomes
HRD-LOH: HRD-loss of heterozygosity
TF: Transcription factors
FDR: False discovery rate
TCGA: The Cancer Genome Atlas
ESTIMATE: Estimation of STromal and Immune cells in MAlignant Tumor tissues using Expression data
ECM: Extracellular matrix
E2F: E2 factor
EGF: Endothelial cells secrete factor
VEGF: Vascular endothelial growth factor
EMT: Epithelial-mesenchymal transition
MCY: Microcystin synthetase
TGF-beta: Transforming growth factor beta
CTLA-4: T-lymphocyte-associated antigen 4
TILs: Tumor-infiltrating leukocytes
GEP: Gene expression profiling
